# Effect of PMMA/Silica Hybrid Particles on Interfacial Adhesion and Crystallization Properties of Poly(lactic acid)/Block Acrylic Elastomer Composites

**DOI:** 10.3390/polym12102231

**Published:** 2020-09-28

**Authors:** Gi Hong Kim, Sung Wook Hwang, Bich Nam Jung, DongHo Kang, Jin Kie Shim, Kwan Ho Seo

**Affiliations:** 1 Korea Institute of Industrial Technology, Korea Packaging Center, Bucheon 14449, Korea; kakamate@kitech.re.kr (G.H.K.); jbn5666@kitech.re.kr (B.N.J.); kangppp@kitech.re.kr (D.K.); 2 Department of Chemical Engineering, Keimyung University, Daegu 42601, Korea; swhwang@kmu.ac.kr; 3 Department of Chemical and Biological Engineering, Korea University, Seoul 02841, Korea; 4 Department of Polymer Science and Engineering, Kyungpook National University, Daegu 41566, Korea

**Keywords:** poly(lactic acid), PMMA/silica hybrid particles, block acrylic elastomer

## Abstract

Poly(lactic acid) (PLA) is a relatively brittle polymer, and its low melt strength, ductility, and thermal stability limit its use in various industrial applications. This study aimed to investigate the effect of poly(methyl methacrylate) (PMMA) and PMMA/silica hybrid particles on the mechanical properties, interfacial adhesion, and crystallization behavior of PLA/block acrylic elastomer. PLA/block acrylic elastomer blends exhibit improved flexibility; however, phase separation occurs between PLA and block acrylic elastomer domains. Valid time-temperature superposition (TTS) measurements of viscoelastic behavior were obtained and exhibited interfacial adhesion with the addition of PMMA or PMMA/silica in PLA/block acrylic elastomer blends. In particular, the phase separation temperature was increased by the incorporation of PMMA/silica hybrid particles, which suggests a potential role for these particles in improving the phase stability. In addition, PMMA inhibits crystallization, while PMMA/silica acts as a nucleating agent, thus increasing the crystallization rate and crystallinity degree.

## 1. Introduction

Poly (lactic acid) (PLA) is a thermoplastic aliphatic polyester that has gained substantial attention due to its potential to substitute for traditional petroleum-based polymers [1,2,3]. However, PLA has a low fracture toughness, ductility, thermal stability, and low melt strength, limiting its use in various industrial applications. Numerous studies have reported on PLA developments, such as copolymerization [4,5] or blending with other polymers or plasticizers [6,7,8]. However, copolymerization is less cost-effective than commercial plastics, and plasticization is limited in product applications due to migration and negative consumer perceptions of plasticizers. Thus, many studies have been conducted to improve the properties of PLA by blending other polymers [9,10,11,12,13,14].

Silica nanoparticles have been used to improve the mechanical properties, adhesion, durability, and wear resistance of polymers. It has a strong polarity due to the presence of the hydroxyl group on the surface, rendering it difficult to disperse as agglomerates in polymer composite preparation. Numerous studies have reported the dispersion of silica nanoparticles in composites via surface pretreatment of modifications [15,16,17,18,19]. Yan et al. [15] reported that a modified L-lactic acid oligomer on the silica surface was dispersed in the PLA matrix, resulting in increased toughness and tensile strength. Zhu et al. [16] reported that oleic-acid-modified silica nanoparticles increased the dispersibility and interfacial adhesion of PLA nanocomposites compared to those of unfilled PLA. Yu et al. [17] used hydrophobic silica particles to increase the impact strength and tensile toughness of PLA/elastomer blends. Lv et al. [19] reported an enhanced mechanical and thermal stability achieved by modifying a silane coupling agent with epoxy on the surface of silica nanoparticles. Vrsaljko et al. [18] prepared composites using silica nanoparticles in PLA/low-density polyethylene (LDPE) blends. Unmodified silica nanoparticles were distributed in PLA and at the PLA interface, but silane-modified silica nanoparticles were present in the LDPE. In addition, silica nanoparticles can improve the compatibility between PLA and LDPE phases, thus increasing the toughness and crystallinity of the final material. Silica nanoparticles stabilize the interface between the matrix and domain, thereby enhancing the compatibility and mechanical properties [17,18,20]. As the literature reports, the crystallization behavior of PLA can be enhanced by using various additives as nucleating agents [16,21,22,23,24,25,26,27,28,29,30,31,32,33,34]. There is a variety of additives including talc [21,22,23], carbon nanotube [24,25], layered silicates [26,27,28], nanocellulose [29], and silica nanoparticles [16,30,31,32,33,34]. The typical additive amount of less than 5 wt.% results in an effective improvement of the nanocomposites. Silica nanoparticles can potentially be used as nucleating agents to increase the crystallinity and crystallization rate of PLA [16,30,31,32,33,34]. In particular, the mechanical properties and crystallinity are improved at a silica content of 2 to 5 wt.% [15,16].

**Scheme 1:**
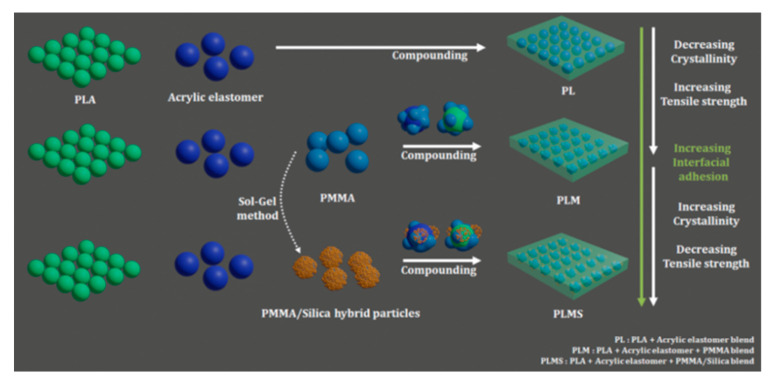
Schematic of the manufacturing and properties of poly (lactic acid) (PLA) composites with block acrylic elastomer, poly(methyl methacrylate) (PMMA), and PMMA/silica hybrid particles.

In this study, we prepared polyvinylpyrrolidone (PVP)-functionalized poly(methyl methacrylate) (PMMA) via emulsion polymerization and used it as a template for adsorption of silica nanoparticles onto the surface via a sol-gel method. As a result, it was possible to obtain raspberry-like PMMA/silica hybrid particles. PMMA/silica hybrid particles reduced the initial agglomeration among silica particles. In addition, these PMMA particles, which are compatible with PLA, were introduced to disperse silica nanoparticles as a carrier system. In addition, the flexibility of PLA was given by using a triblock acrylic elastomer composed of PMMA and poly(n-butyl acrylate) (PnBA). It exhibits high dispersion in the PLA matrix because the PMMA region is compatible with PLA [35,36,37]. The “soft” PnBA region can complement the flexibility of PLA; however, the relatively high PnBA content causes phase separation of the microstructure and viscoelastic behavior [38,39]. Thus, we introduced PMMA and PMMA/silica hybrid particles to increase the interfacial adhesion on PLA/block acrylic elastomer blends. Scheme 1 is shown for the manufacturing process and properties of the PLA composites with PMMA and PMMA/silica hybrid particles. The mechanical, morphology, rheological, and thermal properties were analyzed by a universal testing machine (UTM), field-emission scanning electron microscopy (FE-SEM), rheometer, and differential scanning calorimeter (DSC). It was confirmed by a valid time-temperature superposition (TTS) of viscoelastic behavior that the introduction of PMMA results in phase stability between the PLA matrix and block acrylic elastomer domain. In addition, PMMA/silica hybrid particles increase the phase separation temperature in the low–frequency region. The block acrylic elastomer and PMMA are amorphous polymers that suppress the crystallization behavior of PLA, but silica nanoparticles of PMMA/silica hybrid particles act as a nucleation agent to increase crystallinity and crystallization rate. 

## 2. Materials and Methods

### 2.1. Materials

PLA (PLA4032D) was obtained from Nature Works (USA). Block acrylic elastomer (PMMA-PnBA-PMMA triblock copolymer, LA2330) was obtained from Kuraray (Tokyo, Japan), and primary and secondary antioxidants (SONGNOX 1680, 1010) were purchased from Songwon, Korea. Methyl methacrylate (MMA) and methyl acrylate (MA) as monomers, 1-dodecanethiol as a chain transfer agent (CTA), ammonium hydroxide (NH_4_OH, 28 wt.%) as a catalyst, potassium persulfate (KPS) as an initiator, polyvinylpyrrolidone (PVP, M_w_ ~55,000) as a stabilizer, and tetraethylorthosilicate (TEOS) as a precursor were purchased from Sigma-Aldrich Corp. and were used as received. Deionized water was used in all polymerization and synthesis processes after being purified in an Aquapuri system (551 series, YOUNGIN Scientific, Seoul, Korea)

### 2.2. Preparation of PMMA/Silica Hybrid Particles

**Figure 1:**
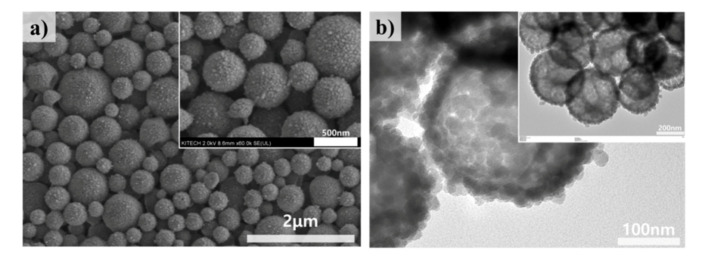
(**a**) SEM and (**b**) TEM images of the PMMA/silica hybrid particles.

The compositions of the PMMA and PMMA/silica hybrid particles are shown in Table 1. PVP-functionalized PMMA particles were prepared via emulsion polymerization. In polymerization, MMA, PVP, KPS, and CTA were added to the reaction medium (methanol/water), stabilized for approximately 30 min, and then polymerized at 60 °C for 24 h. The obtained colloid dispersion solution was washed by several cycles of centrifugation with deionized water. PMMA/silica hybrid particles were synthesized via a sol-gel method. In synthesis, PVP-functionalized PMMA and NH_4_OH were added to the reaction medium (methanol/water), dispersed under stirring and sonicating. TEOS was added at a rate of 2 mL/h and then synthesized at room temperature for 24 h. The obtained colloid dispersion solution was washed by several cycles of centrifugation and re-dispersion. Finally, PMMA/silica hybrid particles were obtained after vacuum freeze-drying. Figure 1 shows the SEM and TEM images of the PMMA/silica hybrid particles. PMMA particles have an average particle size and standard deviation of approximately 716 nm and 0.138, respectively [40]. PMMA/silica hybrid particles have a silica particle size and silica content of approximately 23 nm and 18 wt.%, respectively. All samples were dried for at least 24 h in a vacuum oven. 

### 2.3. Preparation of PLA Composites

Table 2 shows the compositions and sample names of the PLA composites. PL samples were prepared to investigate the effect of the block acrylic elastomer on PLA. PLM and PLMS were fixed with 30 wt.% of block acrylic elastomer, then PMMA was added 0.85, 2.55, 4.25, and 5.95 phr, and PMMA/silica was added 1.00, 3.00, 5.00, and 7.00 phr, respectively. PMMA was composed by calculating about 85 wt.% of PMMA/silica. The PLA composites were mixed at 50 rpm for 10 min at 180 °C by an internal mixer (Brabender Plasti-Coder equipped with a W50 EHT chamber, Brabender GmbH, Duisburg, Germany). For all samples, 0.10 and 0.20 phr of the primary and secondary antioxidants were added. Injection molding was performed with preheating at 180 °C for 4 min (Xplore Micro 10cc Injection Molding Machine, Xplore, Sittard, Netherlands), and the specimens were prepared at a mold temperature of 60 °C.

### 2.4. Universal Testing Machine Analysis

The mechanical properties of the PLA composites were analyzed by a universal testing machine (UTM, Instron 3367, Instron, Norwood, MA, USA). The measurements were performed for a “dog-bone” injection specimen at a cross speed of 10 mm/min and a load cell of 10 kN. The results represent the mean and standard deviation of the five measurements. 

### 2.5. Differential Scanning Calorimetry and Thermal Gravimetric Analysis

DSC (Q2000, TA Instruments, New Castle, DE, USA) was applied to investigate the thermal properties of the PLA composites, including the glass transition temperature (*T*_g_), melting temperature (*T*_m_), crystallization temperature (*T*_c_), and cold crystallinity (*X*_cc_). The crystallinity of the PLA was calculated as the crystallization enthalpy that arises during the heating process because no crystallization enthalpy arises during the cooling process:

(1) $$X_{\mathrm{cc}}\left(\%\right)=\frac{∆H_{c}}{∆H_{m}^{c}}\times 100$$

where *H*LA*_m_* is the melting enthalpy (J/g), *H_c_* is the crystallization enthalpy, and $H_{m}^{c}$ is the melting enthalpy of 100% crystalline P, taken as 93.0 J/g. [32] All tests were performed using a sample weight of 5 ± 0.5 mg under nitrogen gas and maintained to remove the thermal history effect at 220 °C for 3 min. Isothermal/non-isothermal crystallization analysis was conducted to determine the crystallinity and crystallization rate of the PLA composites. For isothermal analysis, the samples were heated to 200 °C, maintained for 5 min, and then rapidly cooled to 110 °C at 50 °C/min, the crystallization behavior was observed over 60 min. Non-isothermal crystallization was measured at a heating rate of 5, 10, and 15 °C/min, respectively. 

The thermal decomposition temperature was measured by a thermogravimetric analyzer (TGA, Q500, TA Instruments, New Castle, DE, USA), and the silica contents were confirmed by the residual amount. Measurements were performed using a weight of 10 ± 0.5 mg to raise the temperature to 800 °C under nitrogen gas. 

### 2.6. Field-Emission Scanning Electron Microscopy Analysis

The morphology of the PLA composites was confirmed by a field-emission scanning electron microscopy (FE-SEM, SU8020, Hitachi, Tokyo, Japan) under an acceleration voltage of 3 kV. SEM images were collected for the side of a fractured tension specimen, a cryo-fractured surface in liquid nitrogen, and a surface extracted in xylene for PLA composites. The surfaces were coated with platinum (Pt) by an ion sputter (E-1045, Hitachi, Tokyo, Japan).

### 2.7. Rheometry Analysis

An oscillation rheometer (Physica MCR302, Anton Paar GmbH, Graz, Austria) was applied to investigate the rheological properties of the PLA composites, including the storage modulus (G′), loss modulus (G″), complex viscosity (|𝜂^*^|), and tan δ. These properties were measured under oscillatory shear on disk specimens with a diameter of 25 mm and a width of 1 mm with parallel plate geometry at 170, 180, 190, 200, 210, and 220 °C, respectively. The analysis was conducted at 0.01 to 100 rad/s with an amplitude of 1%.

## 3. Results

### 3.1. Mechanical Properties

**Figure 2:**
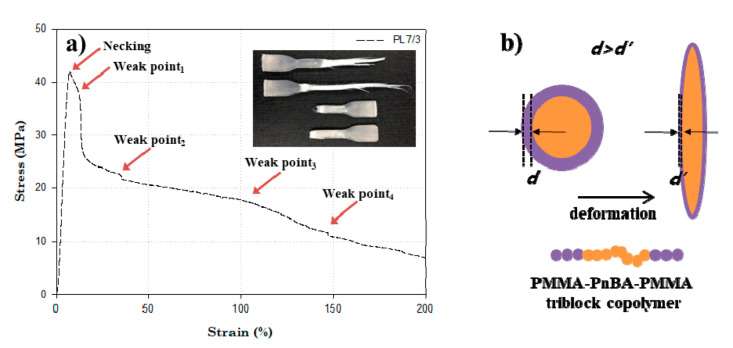
(**a**) Stress-strain curve of PL7/3 and a fractured tensile test specimen. (**b**) Schematic of deformation of the acrylic elastomer domain.

**Figure 3:**
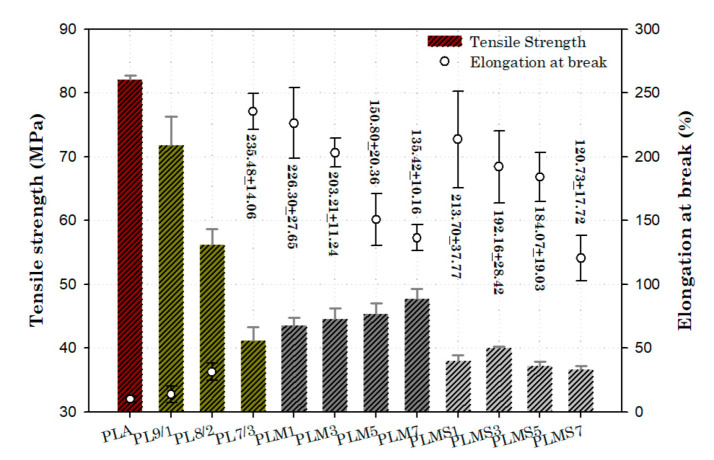
Tensile strength and elongation at the break of neat PLA and PLA composites.

Figure 2a shows a stress-strain curve (S-S curve) and fractured tensile test specimen of the PL7/3. Weak points appear after the yield point, and the stress gradually decreases in the S-S curve. The S-S curve is similar to that of fiber-reinforced composites because the matrix and fibers were separated due to low interfacial adhesion [41]. Similar results were reported for PLA/NR-PMMA/NR blends by Yukun et al. [42]. This result was shown because of the separation between the domain and the matrix during deformation. Figure 2b shows a schematic before and after deformation of the block acrylic elastomer. When deformation occurred due to stress, the PnBA stretched and the area of PMMA per unit volume decreased, resulting in a narrowing of the interfacial distance (*d* > *d*’). Therefore, the interfacial adhesion between PLA and block acrylic elastomer was decreased, resulting in breakage of the matrix like fiber strands during deformation. Figure 3 shows the mechanical properties of the PLA composites. The PL samples decreased the tensile strength and increased the elongation at the break while increasing the block acrylic elastomer contents. In particular, the elongation of PLA composites was greatly increased to about 235% in about 30 wt.% of block acrylic elastomer. The block acrylic elastomer confirmed the composition of approximately 72% PnBA and approximately 28% PMMA by proton nuclear magnetic resonance (^1^H NMR) analysis as shown in Figure S1. PMMA is known to be miscible with PLA, but PnBA has low miscibility [43]. The “hard” PMMA region of the block acrylic elastomer induces dispersion and interfacial adhesion in the PLA matrix, while the “soft” PnBA region exhibits the flexibility of PLA. After fixing to 30 wt.% content, the addition of PMMA increases the tensile strength and decreases the elongation at the break. High interfacial bonding increases the tensile strength of the fiber-reinforced composites [44,45]. This result might be attributed to the compatibility and interfacial adhesion of PLA and PMMA. However, the addition of PMMA/silica decreases the tensile strength and elongation compared to PLM. The decrease in tensile strength might be attributed to the stress concentration effect induced by silica in the “Hard” PLA matrix [46]. The elongation tends to decreases slightly, but it is considered to have a low effect on the “soft” PnBA domain within the range of standard deviation.

### 3.2. Morphology

**Figure 4:**
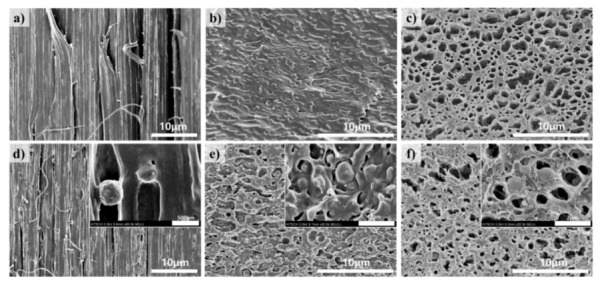
SEM images of (**a**,**d**) the side of a fractured tension specimen, (**b**,**e**) a cryo-fractured surface, and (**c**,**f**) a surface extracted in xylene for (**a**–**c**) PLM7 and (**d**–**f**) PLMS at high magnification.

To verify the miscible between the PLA and the block acrylic elastomer, PMMA, and PMMA/silica particles, SEM analysis was performed to investigate the dispersion and shape of the block acrylic elastomer phase in the PLA matrix. The morphologies of the stretched surface, cryo-fractured surface, and fractured surface extracted in xylene for PLM7 and PLMS7 are shown in Figure 4. The stretched surface separated like a fiber during the tensile test under uniaxial deformation, as shown in Figure 4a,d. As mentioned above, the weak points were attributed to matrix separation from the interface during deformation. In PLMS7, the silica that formed on the PMMA surface was observed as an aggregated shell rather than as individual particles; this trend was observed between the matrix and domain, as shown in Figure 4d,f. It acts as a defect, decreasing the tensile strength of PLA composites. In general, polymer blends can be classified as having a dispersed or co-continuous morphology. The dispersed morphology forms a spherical shape to minimize the free surface energy of the blend system. The morphology exhibits cylindrical shapes because the dispersed particles solidify before regaining an energetically favorable spherical shape [47]. The cryo-fractured surface shows a continuous morphology for PLM7 and PLMS7. In contrast, the fractured surface extracted in xylene shows an island-like morphology. These results show that the PnBA of block acrylic elastomer dispersed in a droplet with low miscibility with PLA, but PMMA with high miscibility appears as a continuous morphology. In Figure S3, the dispersed particle area of PLM7 is approximately 33.3%, based on image analysis, confirming that the block acrylic elastomer and PMMA are removed. Assuming that the particles are circular, the particles were dispersed in a microstructure in the PLA matrix with a diameter of approximately 0.12 to 1.80 µm. PMMA/silica particles are formed to be larger than the acrylic elastomer domain, and thus it is considered that they acted as a defect in mechanical strength. The morphology of PLMS7 indicates that the removal of block acrylic elastomers and PMMA were poorer than that of PLM7. The interfacial adhesion between PLA and the acrylic elastomer is considered to be increased by silica particles. In addition, these results might be because the silica particles in the composites act as anchor segments and prevent the swelling that occurs in the organic region [48].

### 3.3. Rheology

**Figure 5:**
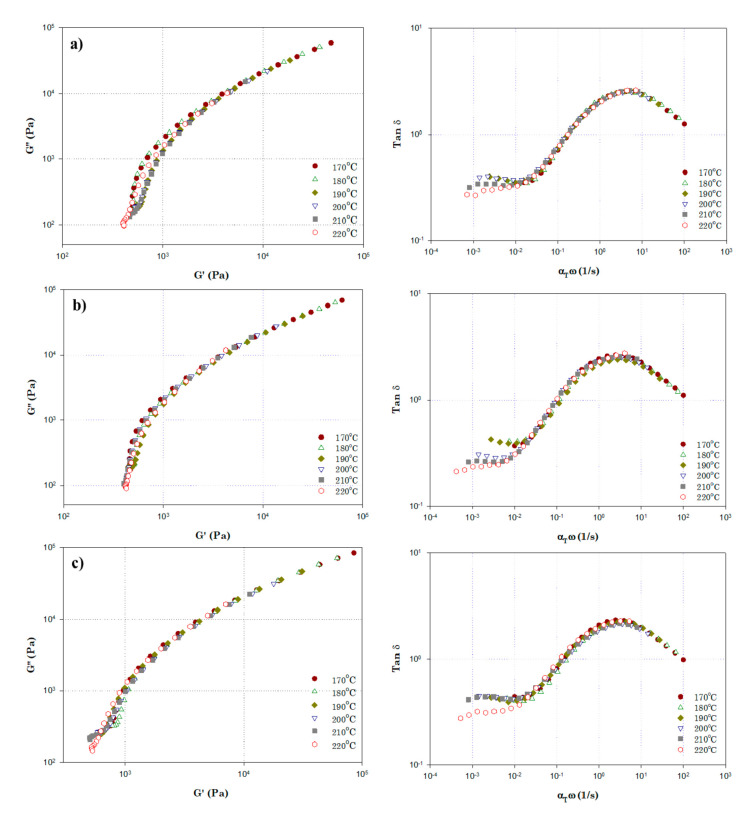
Modified Cole–Cole plots (i.e., log G′ vs. log G″, left) for (**a**) PL7/3, (**b**) PLM7, and (**c**) PLMS7. Master curves of tan δ (right) for (**a**) PL7/3, (**b**) PLM7, and (**c**) PLMS7 over a temperature range of 170–220 °C.

**Figure 6:**
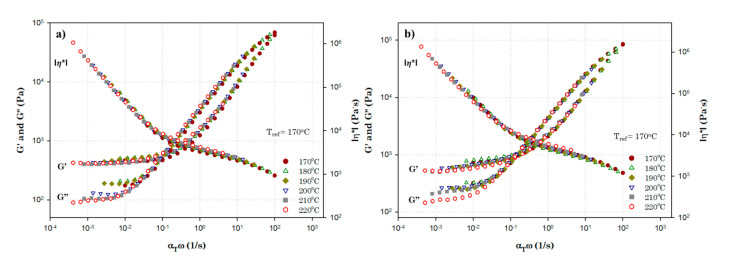
Master curves of G′, G″, and |𝜂^*^| for (**a**) PLM7 and (**b**) PLMS7 for a temperature range of 170–220 °C, where $a_{T}$ is the shift factor for constructing the master curves.

To confirm the validity of the time-temperature superposition (TTS), modified Cole–Cole plots and master curves of tan δ for PL7/3, PLM7, and PLMS7 for a temperature range of 170–220 °C are shown in Figure 5. If vertical factor $b_{T}$ is 1, the relation is independent of the horizontal shift factor. In other words, the plot of the loss modulus (G″) as a function of storage modulus (G′) is independent of temperature [49]. Therefore, the modified Cole–Cole plots would lie on a single curve. PL7/3 did not exhibit a single curve, while PLM7 and PLMS7 displayed single curves, as shown in Figure 5 (left). The master curve of tan δ is shown in Figure 5 (right), with a shift factor of $a_{T}.$ The plots were found to align on a single curve, except in the low-frequency region. The results deviate from the TTS overlap due to the longer heat exposure in the lower frequency region [50]. Figure 6 shows the master curve of G′, G″, and |𝜂^*^| for PLM7 and PLMS7 with a shift factor $a_{T}$. The plots for PL7/3 do not give a valid master curve and thus are not shown in this figure. The TTS is not valid due to the presence of different phases or phase separation [38,51,52,53,54]. In particular, it is known that the linear viscoelastic behavior of the block copolymer is strongly influenced by the phase separation of the microstructure, [38,39] which does not always exhibit TTS in a certain frequency [55,56,57,58]. The addition of PMMA increases the interfacial adhesion between the PLA matrix and the block acrylic elastomer to compensate for phase separation of the microstructure. Silica nanoparticles are also known to inhibit phase separation. Numerous studies have reported on enhanced interfacial adhesion between the matrix and domain due to silica nanoparticles [17,18,20,35]. These particles enhance the compatibility by increasing the interfacial adhesion for PLA/LDPE, [18] PLA/PMMA [35], and PLA/PBAT blends [20]. We can confirm the phase separation temperature in the low-frequency region of the master curve for tan δ, G′, and G″ by using the TTS [35,53]. As shown in Figure 5 and Figure 6, the TTS of PLM7 is valid below 180 °C but exhibits a deviation at higher temperatures. Moreover, PLMS7 exhibits a deviation above approximately 220 °C in the low-frequency region. Therefore, the addition of PMMA suppresses the phase separation of the PLA/block acrylic elastomer blend, and PMMA/silica increases the phase separation temperature, resulting in enhanced phase stability. The TTS of PLA typically has G″ and G′ slopes of 1 and 2, respectively, in the terminal region, where G′ is smaller than G″ [59]. However, in the PLA/block acrylic elastomer blend system, the slopes of G″ and G′ are approximately 0.08–0.10, and G′ is larger than G″ in the terminal region. The results also show a weak dependence on frequency. This viscoelastic behavior is usually seen in network structures. The block acrylic elastomer was dispersed as a microstructure in the PLA matrix to form a network structure of PMMA regions. The system has a relatively high content of PnBA, which results in predominantly elastic properties. 

**Figure 7:**
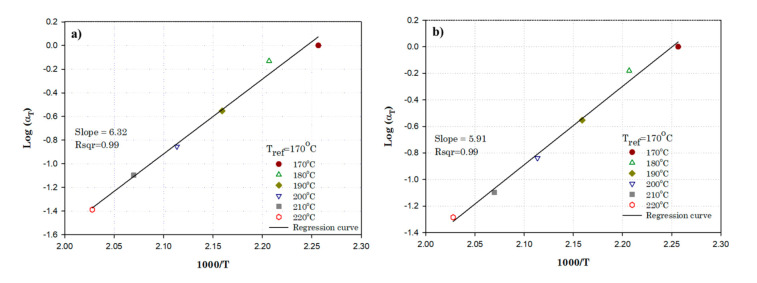
Log (α_T_) as a function of 1/T for (**a**) PLM7 and (**b**) PLMS7 based on the Arrhenius equation.

The temperature dependence of the viscosity is an important factor in polymer flow. The Arrhenius equation for the temperature dependence of viscosity is as follows [49,60,61]:

(2) $$\eta \left(T\right)=A\exp\left(\frac{E_{a}}{RT}\right)$$

(3) $$\log a_{T}=\frac{E_{a}}{2.303R}(\frac{1}{T}-\frac{1}{T_{ref}})$$

where A is the front factor, $E_{a}$ is the activation energy, *R* is the gas constant of 8.315 J/mol∙K, and *T* is the absolute temperature. Figure 7 presents log ($a_{T}$) as a function of 1/T for PLM7 and PLMS7 based on the Arrhenius equation. According to Equation (3), the $E_{a}$ values for PLM7 and PLMS7 are approximately 121.10 kJ/mol and 113.17 kJ/mol, respectively; the *E_a_* represented the effect of the temperature on the behavior of blends. These results indicate that the melting behavior of PLM7 is more sensitive to temperature compared with PLMS7. For comparison, the *E*_a_ of neat PLA, PMMA, and PLA/PMMA 70/30 have been reported in the literature as approximately 80, 182, and 110 kJ/mol, respectively [36,62,63]. That activation energy decreased with the silica particles might be explained by the status that the silanol group on the surface may form a hydrogen bond with the PLA and bring down the energy barrier [64]. Furthermore, the incorporation of silica particles into the PLA matrix induces more heterogeneous nucleation, causing a lower *E_a_* value [65]. 

### 3.4. Thermal Properties

**Figure 8:**
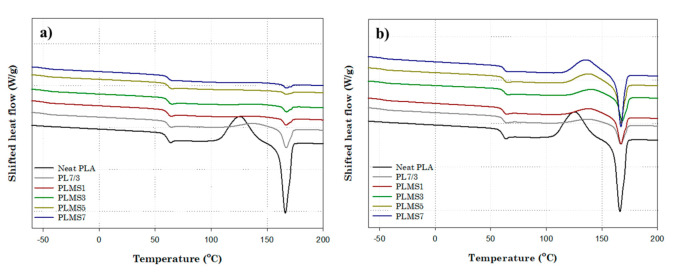
DSC thermogram for neat PLA, PL7/3, and (**a**) PLM samples and (**b**) PLMS samples at a heating rate of 10 °C/min.

Figure 8 and Table 3 present the DSC graphs and thermal properties of the PLA composites at a heating rate of 10 °C/min. The PLA composites have two glass transition temperatures (*T*_g1_*, T*_g2_), approximately −47 and 60 °C, corresponding to the PnBA region of the acrylic elastomer and the PLA region, respectively. Two compatible polymers usually have a single *T*_g_ or two shifted *T*_g_ values that are intermediate between the temperatures for each polymer. Therefore, it is confirmed that the PLA and PnBA region of the block acrylic elastomer are not compatible. However, the addition of PMMA causes a slight increase in the *T*_g_ of PLA from approximately 59.6 to 61.4 °C, and the addition of PMMA/silica increases the temperature to approximately 61.8 °C. The PLMS show a greater increase in *T*_g_ than PLM7, which is influenced by hydrogen bonding of the silica particles. The *T*_g_ of polymer blends is known to be influenced by intermolecular interactions such as hydrogen bonding [66,67]. These results indicate a PMMA and silica interaction with the PLA domain. 

The addition of acrylic elastomers reduces the crystallinity of the PLA from approximately 44.1% to 10.2%, and the addition of PMMA reduces the crystallinity to approximately 1.9% and increases the crystallization temperature (*T*_cc_) from approximately 126.2 to 142.5 °C. In particular, even small amounts of PMMA significantly reduce the crystallinity of PLA than block acrylic elastomer. Therefore, the crystallization of PLA is obviously inhibited by the incorporation of PMMA. These results are ascribed to the dilution effects because PLA requires higher energy to transport to the growth of crystal lamella in a miscible system [68]. However, the addition of PMMA/silica increased the crystallinity to approximately 23.5% and *T*_cc_ decreased to 136.3 °C compared to PLM7. The Δ*H*_cc_ of PLMS increases in the order of PLMS3 < PLMS1 < PLMS5 < PLMS7. The enthalpy provides information on the number of active nuclei [69]. Crystallinity improvement has been reported for silica loadings between 2.5 and 5.0 wt.%, but higher silica loadings (10 and 20 wt.%) are known to reduce crystallinity [15,16,32]. In this experiment, PLMS samples confirmed the silica content of 0.10%, 0.56%, 0.89%, and 0.91% by TGA analysis (Table S1 and Figure S2). Therefore, the silica particles act as nucleating agents in the PLA matrix, increasing crystallinity [16,30,31,32,33].

The crystallization behavior strongly influences the mechanical and thermal properties of semicrystalline polymers. The Avrami equation is generally utilized to determine the crystallization kinetics of crystalline polymers, describing the time-dependent relative crystallinity as follows [70,71,72]:

(4) $$\theta \left(t\right)=1-\exp\left(-k_{a}t^{n}\right)$$

(5) ln[−ln(1−θ(t))]= nln(t)+lnk

where $k_{a}$ is the Avrami constant (crystallization rate constant) and *n* is the Avrami exponent. $k_{a}$ and *n* are temperature-dependent parameters and are specific to a given crystalline morphology, such as spheres, disks, and rods, and nucleation type, such as predetermined or sporadic [72]. 

**Figure 9:**
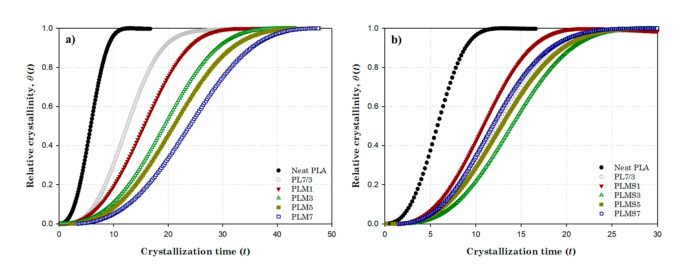
Relative crystallinity (θ(*t*)) vs. crystallization time (*t*) for neat PLA, PL7/3, and (**a**) PLM samples and (**b**) PLMS samples.

**Figure 10:**
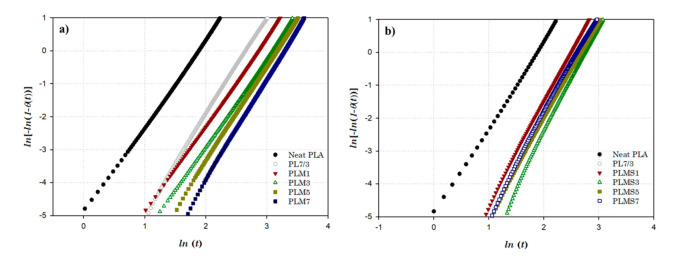
Plot of *ln* [−*ln*(1−*θ*(*t*)) as a function of logarithmic time (*ln*(*t*)) for neat PLA, PL7/3, and (**a**) PLM samples and (**b**) PLMS samples.

In Figure 9, the curves are plotted for the relative crystallinity (θ(*t*)) vs. crystallization time (*t*) of isothermal crystallization at 110 °C. The crystallization half-time (*t*_0.5_) is defined as the time needed to attain 50% crystallinity and can be directly obtained from the curves. Figure 10 presents *ln* [−*ln*(1−*θ*(*t*))] as a function of logarithmic time (*ln*(*t*)). The crystallization parameters are summarized in Table 4. In the PLM samples, the Avrami constant, $k_{a}$, decreases, and *t*_0.5_ increases with increasing PMMA content. The Avrami exponent, *n*, increases from 2.64 to 3.03. The *t*_0.5_ of PLM shows a rapid increase from 12.54 to 23.78 min compared to PL7/3. It can be seen that the addition of PMMA decreases the crystallization rate. PLMS samples have an Avrami exponent *n* of approximately 2.98 to 3.25. It can be expected to have a complete crystalline form. In the PLMS samples, the PLMS1 has the lowest *t*_0.5_, but Δ*t*_0.5_ increases with increasing PMMA/silica content. These results show that PMMA/silica has an effect of inhibiting crystallization due to the PMMA, but the effect of promoting crystallization of silica particles is more largely attributed to it. 

**Figure 11:**
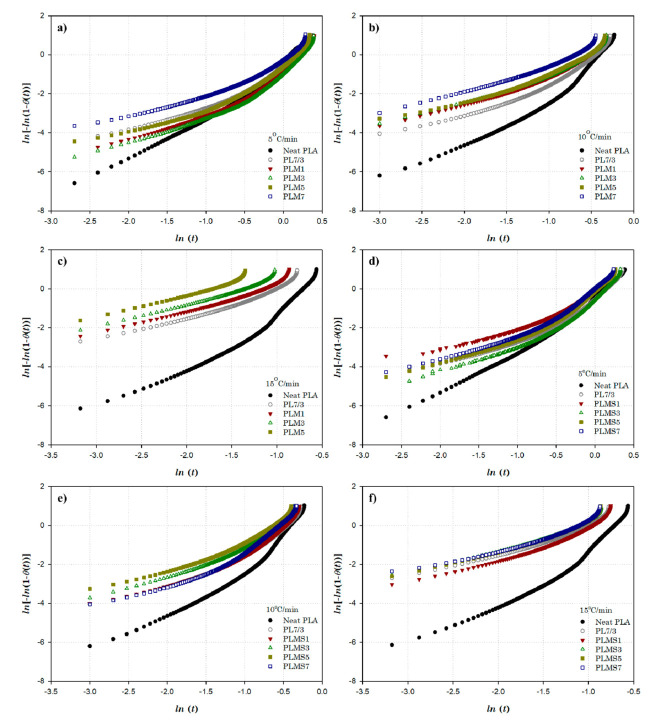
Plot of *ln* [−*ln*(1−*θ*(*t*))] as a function of logarithmic time (*ln*(*t*)) for neat PLA, PL7/3 (**a**,**b**,**c**) PLM samples and (**d**,**e**,**f**) PLMS samples based on the Avrami model for heating rates of 5, 10, and 15 °C/min.

The Avrami equation has been used to describe isothermal crystallization behavior. By assuming that the crystallization temperature is constant, the Avrami equation can also describe non-isothermal crystallization. In a temperature-dependent non-isothermal process, the crystallization rate ($k_{c}$) was modified by Jeziorny as follows [73]:

(6) $$lnk_{c}=\frac{lnk}{\beta }$$

where $k_{c}$ is the modified crystallization rate constant in a non-isothermal process with an assumed heating rate, and β is the heating rate. The crystallization parameters *n* and *k* can be obtained from the slope and intercept in the plot of *ln* [−*ln*(1−*θ*(*t*))] as a function of *ln*(*t*), based on Equations (4) and (5). Figures S4 and S5 and Table S2 show the DSC graph, the relative crystallinity (*θ*(*t*)) vs. crystallization time (*t*), and thermal properties for the PLA composites at heating rates of 5, 10, and 15 °C/min, respectively. Figure 11 displays *ln* [−*ln*(1−*θ*(*t*))] as a function of logarithmic time (*ln*(*t*)) for the PLA composites. According to Equation (5), *n* and *k* can be obtained from the slope and the intercept, and $k_{c}$ can be calculated from Equation (6). The crystallization parameters are summarized in Table 5. In the neat PLA, *n* is approximately 2.0, whereas PL7/3, PLM, and PLMS have values of approximately 1.0–1.3. These results indicate that the non-uniform crystal was formed by one-dimensional crystal growth due to the addition of the block acrylic elastomers and PMMA. The addition of PMMA tends to increase *k*_c_ and decrease *t*_0.5_, while the crystallinity decreases, indicating that the crystallization rate tends to increase with decreasing crystallinity. Moreover, the interactions between the PMMA and PLA molecular chains can disturb the folding of the PLA molecular chains, thus reducing the crystallinity [74,75]. The PLMS does not show a significant difference from PLM, but the crystallinity increases. In addition, it could be seen from the *k*_c_ values that the crystal growth does not have a relatively large difference than that of PLM. Therefore, the addition of PMMA/silica has a greater effect on increasing the number of initial crystal nuclei than increasing the crystal growth rate. 

## 4. Conclusions

In this study, the mechanical properties, viscoelastic behavior, and crystallization of PMMA and PMMA/silica particles in PLA/block acrylic elastomers were investigated. The block acrylic elastomer exhibits a microstructure phase in the PLA matrix and is excellent for enhancing flexibility. Interestingly, the PLA/block acrylic elastomers were transparent blends. However, the interfacial adhesion with PLA was limited by the low composition of PMMA (approximately 28%) compared with PnBA (approximately 72%). Here, the interfacial adhesion was increased by PMMA and PMMA/silica. The results confirm the validity of the TTS. In addition, the PMMA/silica increased the phase separation temperature of the PLA/block acrylic elastomer blends. However, the PMMA/silica remained in the form of a shell and partially acted as an additive defect with regard to the mechanical properties. The block acrylic elastomer and PMMA decreased the crystallization rate and crystallinity of PLA. In particular, a small amount of PMMA significantly decreased the crystallization rate and crystallinity. However, the addition of PMMA/silica has a greater effect on increasing the number of initial crystal nuclei, thereby increasing the crystallization rate and crystallinity. 

## Figures and Tables

**Table 1:** Typical recipes for the preparation of PMMA and PMMA/silica hybrid particles.

**Description**	**MMA (g)**	**MA (g)**	**CTA (g)**	**KPS (g)**	**PVP (g)**	**CH_3_OH (g)**	**H_2_O (g)**
PMMA	20.00	0.40	0.04	0.01	2.00	118.37	59.18
**Description**	**PMMA(g)**	**NH_4_OH(mL)**	**TEOS(mL)**	**CH_3_OH**	**H_2_O**		
PMMA/silica	4.00	5.80	4.00	72.00	4.00		

**Table 2:** Composition and mixing conditions for the PLA composites.

Description	PLA (wt.%)	LA2330 (wt.%)	PMMA (phr)	PMMA/Silica (phr)	Mixing Process
Neat PLA	100.00		-	-	180 °C 50 rpm 10 min
PL9/1	90.00	10.00	-	-	
PL8/2	80.00	20.00	-	-	
PL7/3	70.00	30.00	-	-	
PLM 1, 3, 5, 7	70.00	30.00	0.85, 2.55, 4.25, 5.95		
PLMS 1, 3, 5, 7	70.00	30.00		1.00, 3.00, 5.00, 7.00	

**Table 3:** Thermal properties of PLA composites for a heating rate of 10 °C/min.

Sample	*T*_g1,2_ (°C)	*T*_cc_ (°C)	Δ*H*_cc_ (J/g)	*T*_m_ (°C)	Δ*H*_m_ (J/g)	*X*_cc_ (%)
Neat PLA	59.6	126.2	39.6	166.1	41.0	44.1
PL7/3	−47.5, 60.3	137.9	9.0	167.0	9.5	10.2
PLM1	−46.5, 60.3	138.7	3.1	167.0	3.2	3.5
PLM3	−46.1, 60.7	139.7	2.6	167.4	2.6	2.8
PLM5	−47.8, 61.1	141.7	1.8	167.6	1.8	2.0
PLM7	−47.6, 61.4	142.5	1.7	167.4	1.7	1.9
PLMS1	−47.4, 60.6	138.3	13.2	167.2	13.3	14.3
PLMS3	−46.2, 61.4	140.6	11.3	168.3	11.5	12.3
PLMS5	−46.8, 61.6	138.2	16.7	167.1	16.8	18.1
PLMS7	−47.7, 61.8	136.3	21.8	167.1	21.9	23.5

**Table 4:** Isothermal crystallization parameters for the PLA composites based on the Avrami equation and the crystallization half-time.

Sample	*n*_a_	*ln*(*k*)_a_	*R*_sqr_	*t*_0.5_ (min)	Δ*t*_0.5_^a^ (min)
Neat PLA	2.64	−4.96	0.99	5.73	
PL7/3	2.97	−7.88	0.99	12.54	
PLM1	2.64	−7.58	0.99	15.39	
PLM3	2.71	−8.36	0.99	19.04	
PLM5	2.87	−9.11	0.99	20.91	
PLM7	3.03	−9.98	0.99	23.78	
PLMS1	3.06	−7.65	0.99	10.84	4.55
PLMS3	3.25	−8.97	0.99	14.14	4.90
PLMS5	2.98	−7.99	0.99	12.91	8.00
PLMS7	3.05	−7.95	0.99	12.03	11.75

^a^ Δ*t***_0.5_**is the difference between PLM(*t*_0.5_) and PLMS(*t*_0.5_). (Δ*t***_0.5_** = PLM(*t*_0.5_)−PLMS(*t*_0.5_)).

**Table 5:** Non-isothermal crystallization parameters for the PLA composites based on the Avrami equation and crystallization half-time.

Sample	Rate (°C/min)	*k*_c_	*n*	*t*_0.5_	*X*_cc_ (%)
Neat PLA	5/10/15	0.29/0.55/0.70	2.02/2.01/2.17	5.68/3.60/2.69	42.22/44.13/27.54
PL7/3	5/10/15	0.34/0.65/0.92	1.18/1.22/1.14	6.40/3.23/1.27	28.17/1022/2.17
PLM1	5/10/15	0.28/0.75/0.98	1.28/1.31/1.23	7.93/2.72/0.88	18.63/3.46/1.11
PLM3	5/10/15	0.26/0.76/1.03	1.27/1.26/1.25	8.05/2.68/0.54	12.02/2.75/-
PLM5	5/10/15	0.32/0.77/1.11	1.15/1.27/1.23	6.80/2.61/0.22	8.67/1.98/-
PLM7	5/10/15	0.42/0.84/-	1.00/1.15/-	9.21/1.76/-	7.69/1.85/-
PLMS1	5/10/15	0.43/0.65/0.88	0.97/1.18/1.21	4.64/3.11/1.47	29.98/14.25/4.51
PLMS3	5/10/15	0.29/0.72/0.95	1.20/1.22/1.16	6.27/2.44/1.01	29.32/12.33/2.88
PLMS5	5/10/15	0.34/0.76/0.94	1.16/1.15/1.18	4.91/2.10/1.02	28.80/18.09/4.05
PLMS7	5/10/15	0.37/0.65/0.94	1.13/1.25/1.09	4.46/2.55/1.01	29.18/23.54/4.59

## Supplementary Materials

The following are available online at https://www.mdpi.com/2073-4360/12/10/2231/s1, Figure S1: 1H NMR spectra of the block acrylic elastomer in CDCl3 solvent, Figure S2: TGA curves of neat PLA and PLA composites: (a) neat PLA and PLM and (b) PLMS samples, Figure S3: Morphology of the PLM7: (a) SEM image and (b) image analysis, Figure S4: DSC thermograms for (a) neat PLA, (b) PL7/3, (c) PLM7, and (d) PLMS7 at a heating rate of 5, 10, and 15 °C/min, Figure S5: Relative crystallinity (θ(t)) vs. crystallization time (t) for neat PLA, PL7/3, (a, c, e) PLM, and (b, d, f) PLMS samples at heating rates of 5, 10, and 15 °C/min, Table S1: Thermal degradation properties of neat PLA and PLA composites and residue, Table S2: Non-isothermal properties of the PLA composites at a heating rate of 5, 10, and 15 °C/min.
