# Supplementary material for: Effect of PMMA/Silica Hybrid Particles on Interfacial Adhesion and Crystallization Properties of Poly(lactic acid)/Block Acrylic Elastomer Composites
Source: Polymers (Basel). 2020 Sep 28;12(10):2231. doi: 10.3390/polym12102231 (PMC7650821; doi:10.3390/polym12102231)
Supplement: Supplementary file 1 [file polymers-12-02231-s001.pdf]

Effect of PMMA/Silica Hybrid Particles on Interfacial Adhesion and Crystallization Properties of Poly(lactic acid)/Block Acrylic Elastomer Composites

Gi Hong Kim [1], Sung Wook Hwang [2], Bich Nam Jung [1,3], DongHo Kang [1], Jin Kie Shim [1,*] and Kwan Ho Seo [4,*]

[1] Korea Institute of Industrial Technology, Korea Packaging Center, Bucheon 14449, Korea; kakamate@kitech.re.kr (G.H.K.); jbn5666@kitech.re.kr (B.N.J.); kangppp@kitech.re.kr (DH.K.)
[2] Department of Chemical Engineering, Keimyung University, Daegu 42601, Korea; swhwang@kmu.ac.kr (S.W.H)
[3] Department of Chemical and Biological Engineering, Korea University, Seoul 02841, Korea
[4] Department of Polymer Science and Engineering, Kyungpook National University, Daegu 41566, Korea
* Correspondence: jkshim@kitech.re.kr (J.K.S.); khseo@knu.ac.kr (K.H.S.)

Proton nuclear magnetic resonance (^{1}H NMR) of the block acrylic elastomer

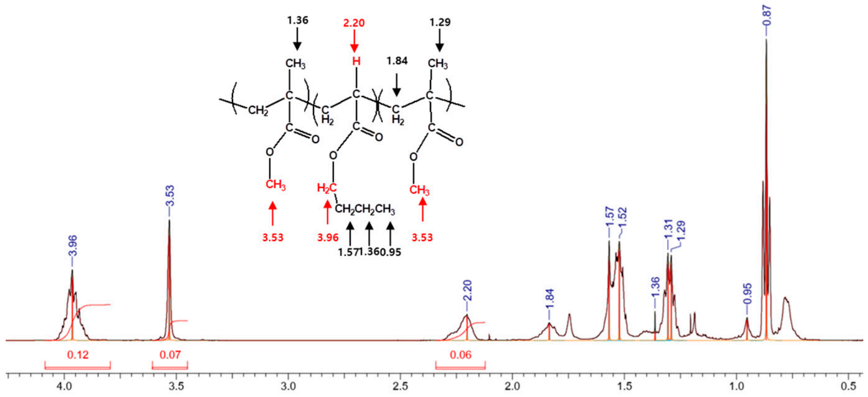

Figure S1. ^{1}H NMR spectra of the block acrylic elastomer in $CDCl_3$ solvent.

The spectra were confirmed at 3.53ppm (O-CH_3, PMMA) and 3.96 ppm (O-CH_2, PnBA). The PMMA and PnBA compositions were calculated from the $PMMA_{3.53ppm}$ and $PnBA_{3.96ppm}$ peak integral values by using the following equations:

$$x + y = 1$$

$$3x_{OCH_3} : 2y_{OCH_2} = (PMMA_{3.53ppm}) : (PnBA_{3.96ppm})$$

where x, and y are PMMA and PnBA mole fractions, respectively. Following this approach, the PMMA and PnBA contents were found to be approximately 28% and 72%.

Thermal gravimetric analysis (TGA) of the PLA and PLA composites

Figure S2 and Table S1 show the thermal degradation properties of neat PLA and PLA composites. The PLA composites with PMMA/silica hybrid particles exhibit slightly enhanced thermal decomposition properties, but the particles are considered to have a minor effect. The silica content was similar to the expected value based on the residual amount.

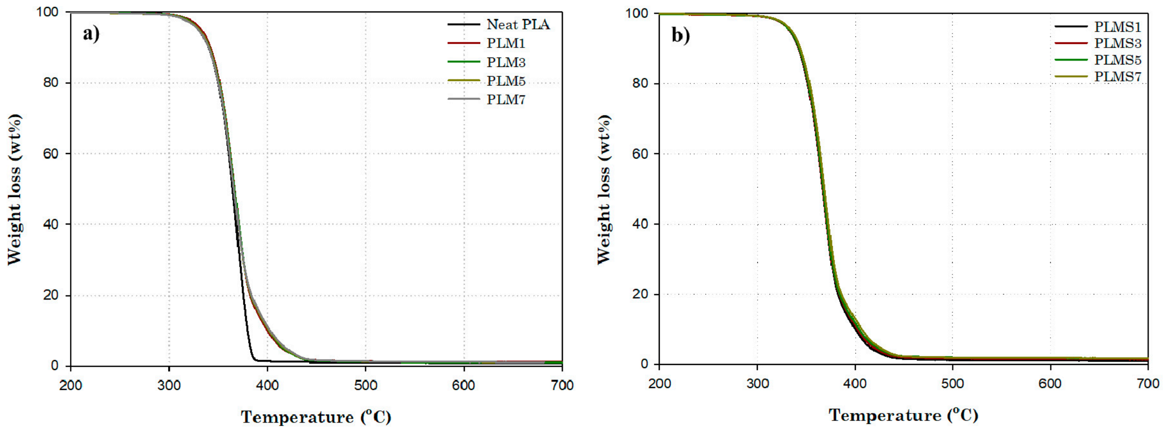

Figure S2. TGA curves of neat PLA and PLA composites: (a) neat PLA and PLM and (b) PLMS samples.

Table S1. Thermal degradation properties of Neat PLA and PLA composites and residue

Sample	T_{10} (°C)	T_{50} (°C)	Residue (wt.%)	Silica content (wt.%)
Neat PLA	339.93	364.44	0.82	-
PLM1	341.85	366.66	1.07	-
PLM3	341.04	366.52	0.90	-
PLM5	340.58	365.83	1.06	-
PLM7	340.12	365.76	1.08	-
PLMS1	342.05	366.68	1.00(0.17)[a]	0.1[b]
PLMS3	342.39	366.87	1.46(0.51)	0.56
PLMS5	342.81	367.38	1.79(0.85)	0.89
PLMS7	343.93	366.80	1.81(1.19)	0.91

a: calculated theoretical silica content(wt.%), b : TGA analysis

Morphological analysis of the PLA and PLA composites

Figure S3 shows a SEM image and transformed image of the PLM7 morphology. The dispersed morphology occupied by each acrylic elastomer was calculated using software, and the occupied area was taken as a circle to enable a calculation of the diameter. Here, to reduce the error, areas of less than 0.01 um[2] were neglected.

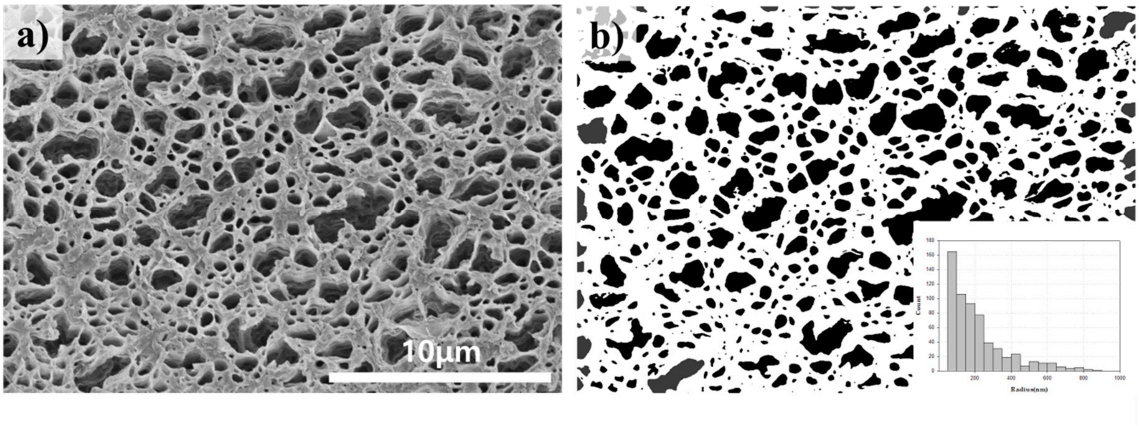

Figure S3. Morphology of the PLM7: (a) SEM image and (b) image analysis and particle size distribution histogram.

DSC analysis of the PLA and PLA composites

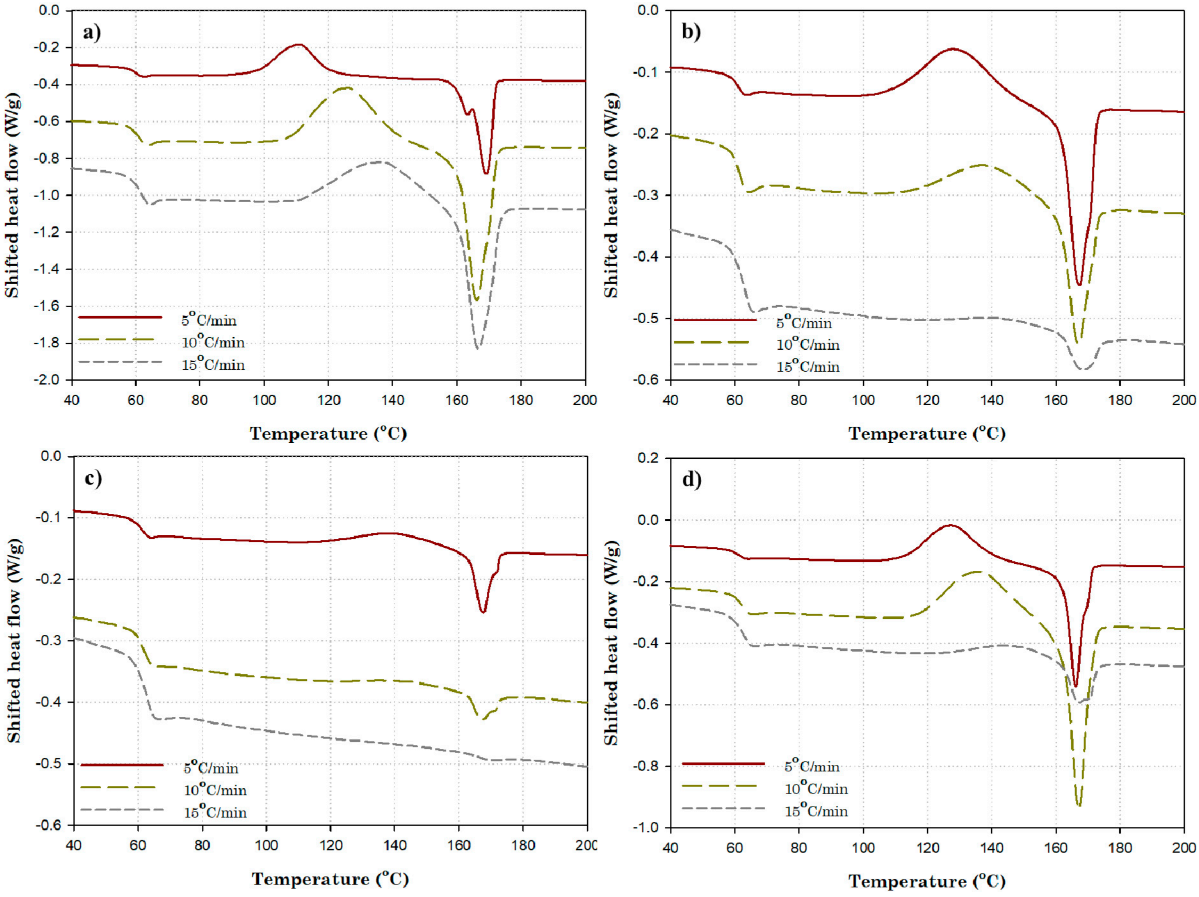

Figure S4. DSC thermograms for (a) neat PLA, (b) PL7/3, (c) PLM7, and (d) PLMS7 at heating rates of 5, 10, and 15°C/min.

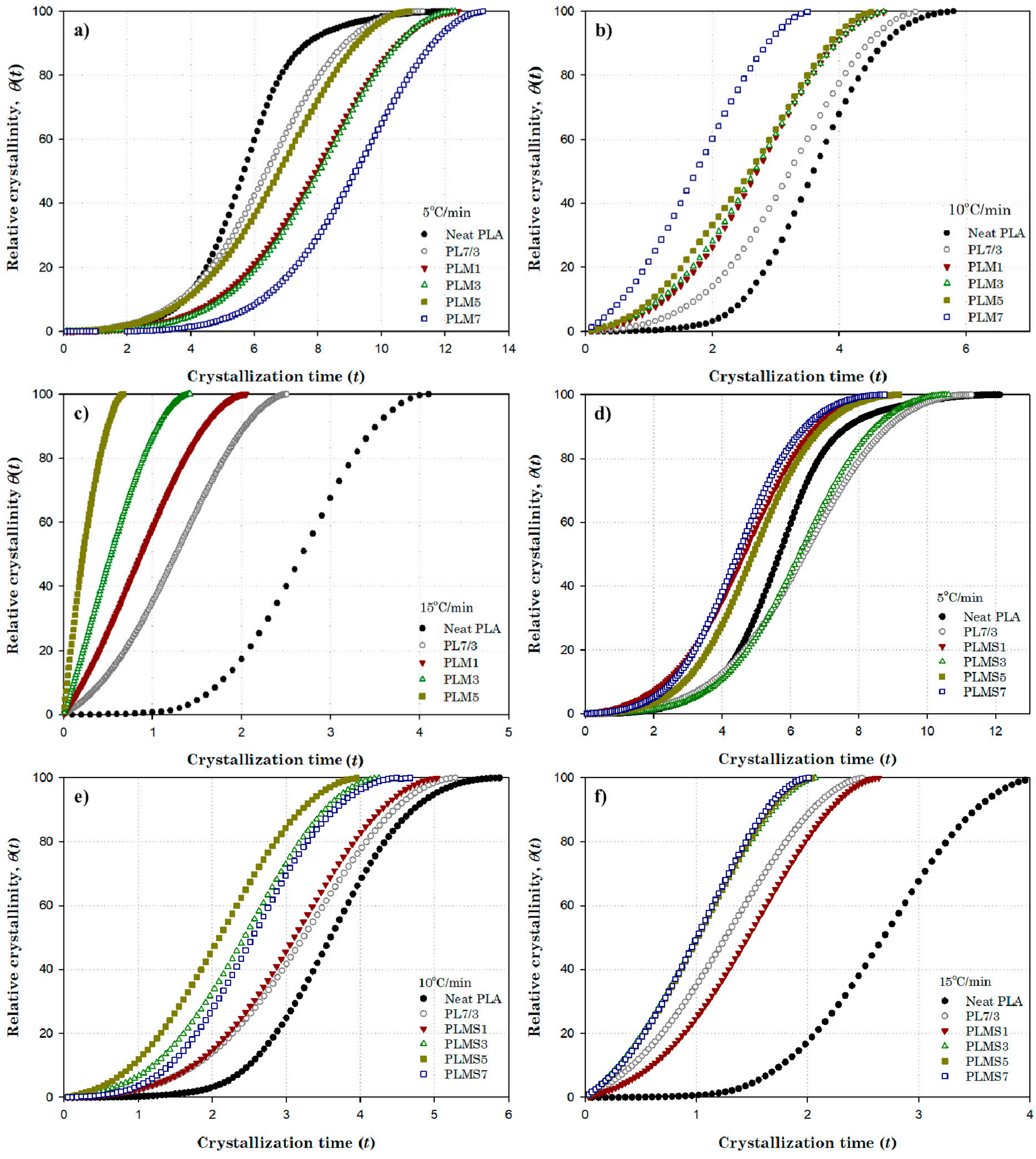

Figure S5. Relative crystallinity ($\theta(t)$) vs. crystallization time (t) for neat PLA, PL7/3, (a, b, c) PLM and (d, e, f) PLMS samples at heating rates of 5, 10, and 15°C/min.

Table S2. Non-isothermal properties of the PLA composites at heating rates of 5, 10, and 15°C/min.

Sample	Rate (°C/min)	T_{cc} (°C)	ΔH_{cc} (J/g)	T_m (°C)	ΔH_m (J/g)	X_{cc} (%)
Neat PLA	5	110.94	34.49	169.20(162.90)	39.26	42.22
	10	126.22	39.59	166.14	41.04	44.13
	15	136.91	24.76	166.71	25.61	27.54
PL7/3	5	128.13	25.40	167.37	26.20	28.17
	10	137.88	9.04	167.04	9.50	10.22
	15	141.66	1.66	168.33	2.02	2.17
PLM1	5	134.90	16.71	162.05	17.322	18.63
	10	138.69	3.05	167.04	3.22	3.46
	15	140.12	0.97	167.10	1.03	1.11
PLM3	5	137.28	10.90	167.41	11.18	12.02
	10	139.69	2.55	167.36	2.56	2.75
	15	142.97	-	168.28	-	-
PLM5	5	138.45	8.00	167.56	8.06	8.67
	10	141.70	1.82	167.59	1.84	1.98
	15	145.17	-	169.47	-	-
PLM7	5	140.05	6.58	167.74	7.15	7.69
	10	142.51	1.72	167.39	1.72	1.85
	15	147.16	-	169.22	-	-
PLMS1	5	125.03	26.58	165.84(170.12)	27.88	29.98
	10	138.34	13.16	167.15	13.25	14.25
	15	141.59	3.59	166.84	4.19	4.51
PLMS3	5	129.27	26.49	166.52	27.27	29.32
	10	140.58	11.34	168.33	11.47	12.33
	15	143.62	2.34	167.06	2.68	2.88
PLMS5	5	127.46	26.60	166.23	26.78	28.80
	10	138.20	16.69	167.05	16.82	18.09
	15	144.28	3.44	167.06	3.77	4.05
PLMS7	5	127.35	26.70	166.21	27.14	29.18
	10	136.32	21.79	167.11	21.89	23.54
	15	145.66	3.77	167.05	4.27	4.59
